# Expression of miR‐634 in gastric carcinoma and its effects on proliferation, migration, and invasion of gastric cancer cells

**DOI:** 10.1002/cam4.1204

**Published:** 2018-02-21

**Authors:** Jiao Guo, Chun‐Dong Zhang, Jia‐Xiang An, Yun‐Yun Xiao, Shuai Shao, Nuo‐Ming Zhou, Dong‐Qiu Dai

**Affiliations:** ^1^ Department of Gastrointestinal Surgery the Fourth Affiliated Hospital of China Medical University Shenyang 110032 China; ^2^ Department of Obstetrics and Gynecology the Shengjing Affiliated Hospital of China Medical University Shenyang 110004 China; ^3^ Cancer Center the Fourth Affiliated Hospital of China Medical University Shenyang 110032 China

**Keywords:** Gastric cancer, *JAG1*, MGC‐803, miR‐634, SGC‐7901

## Abstract

This study aims to observe the expression of microRNA (miR)‐634 in different gastric cancer cell lines and tissues, and to study the effects of miR‐634 on the proliferation, migration, and invasion of the gastric cancer cells. The miR‐634 mimics and miR‐634 inhibitors were transfected by lentivirus into human gastric cancer SGC‐7901 and MGC‐803 cells, and the miR‐634 cells without transfection were used as the control group (NC group). The expression of miR‐634 in the transfected cells was detected by qRT‐PCR. Cell viability was measured by the CCK8 assay. The migration and invasion ability of the cells were detected by scratch assays and Transwell^®^ chamber assays, respectively, and the luciferase assay verified the binding of miR‐634 to the target gene *JAG1*. The expression level of miR‐634 in gastric cancer tissues and cell lines was significantly lower than that in normal adjacent tissues and control cells. The survival of cells was significantly decreased, and number of cells migrating and invading was decreased in the miR‐634 mimics group. However, in the miR‐634 inhibitor group, the opposite results were observed. Over‐expression of miR‐634 inhibited the proliferation, migration, and invasion of gastric cancer cell lines, and the miR‐634 target gene was *JAG1*.

## Introduction

Gastric cancer is one of the most common malignant tumors, and is a serious threat to human health. Gastric cancer accounts for one‐third of cancers worldwide, with nearly 1 million new cases occurring each year 1. In recent years, although diagnosis and treatment options are progressing, the percentage of early diagnosis of gastric cancer remains low, and most patients first diagnosed with gastric cancer are already in the advanced stage or late stage, with local infiltration and lymph node metastasis. Therefore, the overall prognosis is poor 2. It is, therefore, urgent to study the mechanism of gastric cancer progression and metastasis, and to seek effective interventions to improve the prognosis of gastric cancer.

The microRNA (miRNA) is a class of noncoding endogenous small RNAs with lengths of about 18–22 nucleic acids. The miRNAs can combine with mRNA at the 3′‐untranslated region (UTR), thus playing a role in regulating gene expression at the posttranscriptional level 3. It has been reported that miRNAs can be used as oncogenes or tumor suppressors to regulate the proliferation, invasion, and metastasis of tumor cells, inhibit angiogenesis and inhibit cell apoptosis 3, 4, 5, 6. It was also reported that hypermethylation of CpG islands in the promoter region of miRNAs is one of the mechanisms of miRNA silencing in tumors 3, 7, 8. CpG island methylation is tissue and cell specific, is related to the occurrence and development of disease, and is involved in the expression of DNA mismatch repair genes 9.

Studies have indicated that miR‐634 plays an important role in some cancers. For example, miR‐634 inhibited cell growth in breast cancer, but also blocked the HER2 pathway 10. Research also demonstrated that due to the metabolic effects of miR‐634 bicarbonate, it plays an important role in the pathogenesis of obesity 11. In esophageal squamous cell carcinoma, miR‐634 activated the mitochondrial apoptotic pathway and enhanced the cytotoxicity induced by chemotherapy 12. Overexpression of miR‐634 promoted cell apoptosis in ovarian carcinoma, and the expression levels of miR‐634 affected the sensitivity to chemotherapeutic drugs. For example, miR‐634 enhanced the drug sensitivity to cisplatin, carboplatin, and doxorubicin, but did not enhance chemosensitivity to paclitaxel 13. In nasopharyngeal carcinoma, miR‐634 enhanced the drug sensitivity to paclitaxel and reversed Taxol resistance 14. However, there is no report regarding the role of miR‐634 in gastric cancer. As previously suggested, the different expression levels of miR‐634 in different types of cancer are tissue and organ specific. Therefore, we investigated the expression of miR‐634 in gastric carcinomas and its effect on the function of gastric cancer cells.

## Materials and Methods

### The specimens and cell lines

All human gastric cancer cell lines, MGC803, SGC7901, MKN45, HGC27, and the human normal gastric epithelial cell line (GES‐1) were purchased from Shanghai Institute of Cell Biology, Chinese Academy of Sciences (Shanghai, China). All patient tumor and adjacent tissues were obtained from the Fourth Affiliated Hospital of China Medical University from January 2014 to January 2016. Patients were treated with radical gastrectomy for gastric cancer in a total of 83 cases. Primary gastric cancer and adjacent tissues were obtained from each patient, and no patient was not treated with radiotherapy and/or chemotherapy before the operation. Some of the resected specimens were stored in liquid nitrogen prior to DNA and RNA extractions. The study was approved by the medical ethics committee of the Fourth Affiliated Hospital of China Medical University, and all patients provided informed consent.

### Cell culture and demethylation treatment

The cell culture medium used was RPMI‐1640 with 10% fetal bovine serum (Gibco BRL, Grand Island, NY, USA), and the cells were incubated with 5% CO_2_, at 37°C. The changes of miR‐634 expression in gastric cancer cells were observed by demethylation treatment. The demethylation involved 3 mol/L of 5‐aza‐2 deoxycytidine (5‐aza‐d C) added to the cells for 72 h. Dimethyl sulfoxide (DMSO) treatment was used for the control group.

### Quantitative real‐time (RT) PCR

Trizol^™^ was used to extract total RNA (Takara, Tokyo, Japan) from cells and tissues. According to the manufacturer's instructions, cDNA was synthesized by a RevertAid^™^ First Strand Synthesis Kit (Thermo Fisher, Scotts Valley, CA, USA). U6 was used as a reference, and the cDNA template was placed in an Exicycler^™^ 96 real‐time quantitative fluorescence PCR instrument (Bioneer, Daegeon, Republic of Korea). The reactions were as follows: Hsa‐miR‐634 forward, 5′‐CAGTCTCAAACCAGCACC‐3′; reverse, 5′‐TATGGTTGTTCACGACTCCTTCAC‐3′. The PCR thermal cycle parameters were: 95°C, 5 min, 95°C, 30 sec, 55°C, 30 sec, 72°C, 45 sec, and 35 cycles, 72°C, 5 min. The fluorescence signal was collected and the relative expression of mRNA was calculated by the 2^−ΔΔCT^ method.

### Methylation‐specific PCR

MiR‐634 gene methylation and non‐methylation primers were designed by Methprimer software, and methylation‐specific PCR (MSP) detection was performed. The Has‐miR‐634 gene methylation primers were: upstream, 5′‐ATTATGTTAGTTAGGATGGTTTCGA‐3′, downstream, 5′‐ ATATCCACAAACAAATAACTTCGTT‐3′; non‐methylated primers: upstream, 5′‐ATTATGTTAGTTAGGATGGTTTTGA‐3′; downstream, 5′‐ ATATCCACAAACAAATAACTTCATT‐3′. The PCR reaction conditions were as follows: 95°C, 10 min, 94°C, 45 sec, 56°C, 45 sec, 59°C, 45 sec, 72°C, 45 sec, 35 cycles, 72°C, 10 min. The PCR product (2 *μ*L) was separated by agarose gel electrophoresis, stained with ethidium bromide, and directly observed under UV light. Each sample was tested three times to verify the reproducibility of the experiment.

### Lentivirus production and transduction

The construction of the lentivirus vector was performed by GenePharma (Shanghai, China). According to the lentiviral transfection instructions, recombinant miR‐634 lentivirus particles, LV‐hsa‐miR‐634‐inhibition, and LV‐hsa‐miR‐634, were infected into MGC‐803 and SGC‐7901 cells, respectively, and a group of cells without any treatment was established as the control group.

### Cell wound scratch assay

SGC‐7901 and MGC‐803 cells transfected with miR‐634 mimics and miR‐634 inhibitor as well as negative controls were cultured in six‐well plates for 24 h. Then, after using a 200‐*μ*L pipette tip to make a scratch line across the cells, the plates were incubated in 5% CO_2_, at 37°C. Wound healing was observed using an inverted microscope at 0, 24, 48, and 72 h.

### Transwell^®^ migration assay

After transfection, the cells were adjusted to 1 × 10^9^ cells/L, with 200 *μ*L of cells placed in each well on the Matrigel^™^ precoated Transwell^®^ plates in the upper chamber, and with 20% fetal bovine serum and 600 *μ*L of RPMI‐1640 cell culture medium in the lower chamber. After 48 h of culturing, the Transwell^®^ membranes were fixed with 4% paraformaldehyde, stained with Crystal Violet for 10 min, and washed with phosphate‐buffered saline (PBS) three times. The cells that invaded the membrane were observed under a microscope, and the cell invasion was calculated from randomly photographed areas. The cell invasion percentage = (number of invasive cells in each group/untreated control group) × 100%.

### CCK8 assay

Cell proliferation was measured by the CCK‐8 assay, every 24 h. The cells were seeded into 96‐well plates, with 5000 cells per well, 100 *μ*L medium, and 10 *μ*L of the CCK‐8 reagent from the kit. Two hours before the assay of each well, 10 *μ*L of CCK‐8 reagent was incubated with the cells for 2 h at 37°C. The results were measured using a microplate reader at 450 nm (optical density, OD). The relative proliferation activity = treatment group OD/blank control group OD.

### Plate clone assay

After infection, the cells were grown to prepare the cell suspension and diluted suspension. The cells were inoculated into six‐well plates, with 2 × 10^3^ cells/well. Each experimental sample used three wells. The cells were cultured for 2–3 weeks. When the cells colonies were visible to the naked eye, the cell culture was terminated, cells were fixed with methanol, Giemsa stained for 10–30 min, and counted under a microscope. The clone formation percentage (%) = clone number/number of inoculated cells × 100%.

### Apoptosis assay

Gastric cancer cells (1 × 10^6^ cells) were transfected with miR‐634 mimics or inhibitor in six‐well plates. After 72 h, the cells were collected, washed, fixed, and permeabilized. Annexin V‐FTIC/PI (KeyGen, Nanjing, China) was used to visualize the cells, according to the manufacturer's instructions, and flow cytometry was used to determine cell apoptosis.

### Luciferase reporter assay

H‐293T, SGC‐7901, and MGC‐803 cells were seeded into the 24‐well plates for 24 h before transfection. The plasmid was constructed by Genomeditech (Shanghai, China). The Renilla reference plasmid, miR‐634 control cells, miR‐634 mimics cells, and the target gene 3′‐UTR reporter gene plasmid were cotransfected. Luciferase activity was measured using a double luciferase gene reporter system after transfection with Lipofectamine^®^ 2000 (Thermo Fisher) for 48 h.

### Western blotting

RIPA buffer (Vazyme, Nanjing, China) was used to extract total protein. The extraction method was according to the manufacturer's instructions. Protein concentration was determined using the BCA protein concentration assay kit (Takara, Dalian, China). Thirty micrograms of protein was loaded onto SDS‐PAGE gels (Takara) and transferred to a polyvinylidene fluoride membrane (Takara). The membrane was completely immersed in 5% bovine serum albumin (BSA) for 2 h, at room temperature, and the primary antibody was added and incubated at 4°C overnight. The second antibody was then added to the membrane and incubated at room temperature for 2 h. The membranes were visualized using a chemiluminescence system.

### Statistical analysis

All statistical analyses were performed using the SPSS 20.0 software (SPSS Inc., Chicago, IL, USA). The measurement data were expressed by mean ± SD. Pearson's *χ* ^2^‐test was used to analyse the clinicopathologic features. Survival curves were analyzed by Kaplan–Meier method and compared by the log‐rank test. All *P* values <0.05 were considered statistically significant.

## Results

### MiR‐634 was downregulated in gastric cancer tissues and cells

**Figure 1:**
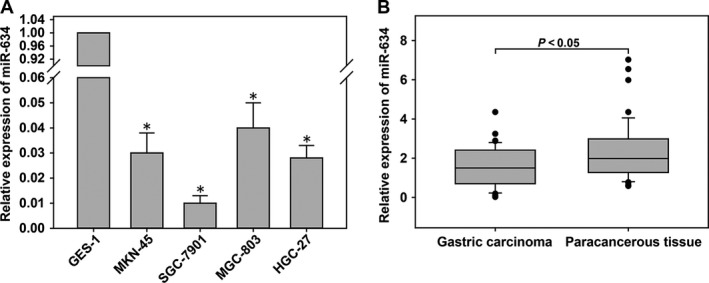
*MiR‐634* was downregulated in gastric cancer (GC) tissues and cells. (A) The expression levels of miR‐634 in GC cells and GES‐1 cells. (B) The expression levels of miR‐634 in 83 pairs of human GC tissues and adjacent normal tissues measured by quantitative real‐time PCR (qRT‐PCR). *, P < 0.05

The expression levels of miR‐634 in the gastric cancer cell lines, HGC‐27, MKN‐45, SGC‐7901, MGC‐803, and the normal gastric epithelial cell line, GES‐1, were detected by quantitative real‐time PCR (qRT‐PCR). Compared with the expression of miR‐634 in normal gastric epithelial cells (GES‐1), the expression of miR‐634 was downregulated in gastric cancer cell lines (Fig. 1A). In addition, the expression level of miR‐634 in 83 gastric cancer tissues and adjacent tissues was detected by qRT‐PCR. The expression level of miR‐634 in cancer tissues was significantly lower than that in the adjacent tissues (Fig. 1B). We also analyzed the correlation between the expression level of miR‐634 and clinical pathological features. The patients were divided into two groups. The cancer tissues with higher than the median expression of miR‐634 were selected as the high group, while those with less than the median expression of miR‐634 were selected as the low group. As shown in Table 1, miR‐634 expression was downregulated significantly in tumors with diameters >3 cm (*P *=* *0.029).

**Table 1:** Expression of miRNA‐634 and JAG1 in human gastric cancer according to patients' clinicopathological characteristics. *, P < 0.05

Characteristics	Number (%)	miR‐634 expression		*P*‐value	JAG1 expression		*P*‐value
		Low group	High group		Low group	High group	
Age (years)							
<60	32	15	17	0.498	12	20	0.496
≥60	51	29	22		24	27	
Gender							
Male	64	34	30	1.000	27	37	0.794
Female	19	10	9		9	10	
Size (cm)							
<3	38	15	23	0.029*	11	27	0.026*
≥3	45	29	16		25	20	
Histology grade							
Well‐moderately	25	12	13	0.634	12	13	0.634
Poorly‐signet	58	32	26		24	34	
Stage							
I/II	28	13	15	0.487	12	16	1.000
III/IV	55	31	24		24	31	
T grade							
T1 + T2	27	17	10	0.246	11	16	0.815
T3 + T4	56	27	29		25	31	
Lymph node metastasis							
Absent(N0)	31	17	14	0.824	15	16	0.501
Present (N1–N3)	52	27	25		21	31	

### The *miR‐634* gene was highly methylated in gastric cancer cell lines and cancer tissues

**Figure 2:**
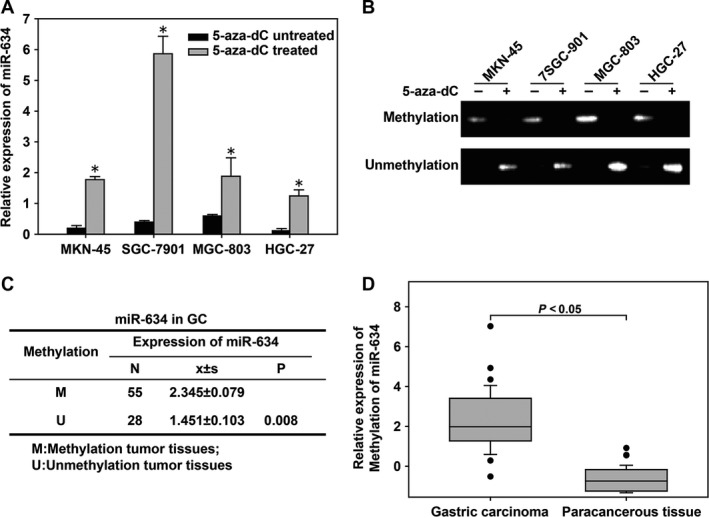
The *miR‐634* gene was highly methylated in gastric cancer cell lines and cancer tissues. (A) Quantitative real‐time PCR (qRT‐PCR) was used to detect the expression of the *miR‐634* gene in gastric cancer (GC) cell lines treated or untreated with 5‐aza‐2 ‐deoxycytidine (5‐aza‐d C). (B) The methylation‐specific PCR (MSP) method was used to detect the methylation status of the *miR‐634* gene in gastric cancer cell lines treated or untreated with 5‐aza‐d C. −, 5‐aza‐d C untreated; +, 5‐aza‐d C treated. (C and D) The relationships between methylation status and expression of *miR‐634* in GC tumor tissues. *, P < 0.05

MSP was used to detect the methylation status of gastric cancer and cancer tissues. The expression of *miR‐634* in gastric cancer cells was relatively low without 5‐aza‐d C treatment, and 5‐aza‐d C could reverse the methylation of *miR‐634* to restore its expression (Fig. 2A). In addition, the gastric cancer cells showed high methylation without 5‐aza‐d C treatment. After 5‐aza‐d C treatment, the gastric cancer cell lines showed a low methylation status (Fig. 2B), suggesting that aberrant methylation of the promoter region of the *miR‐634* gene was an important mechanism leading to its loss of expression in gastric cancer cells. The methylation status of the *miR‐634* gene in gastric cancer and adjacent tissues was determined by the MSP method. The results showed that the methylation of the *miR‐634* gene promoter in gastric cancer tissues was significantly higher than that in adjacent tissues (Fig. 2C and D).

### MiR‐634 inhibited the proliferation, invasion, and migration of gastric cancer cells

**Figure 3:**
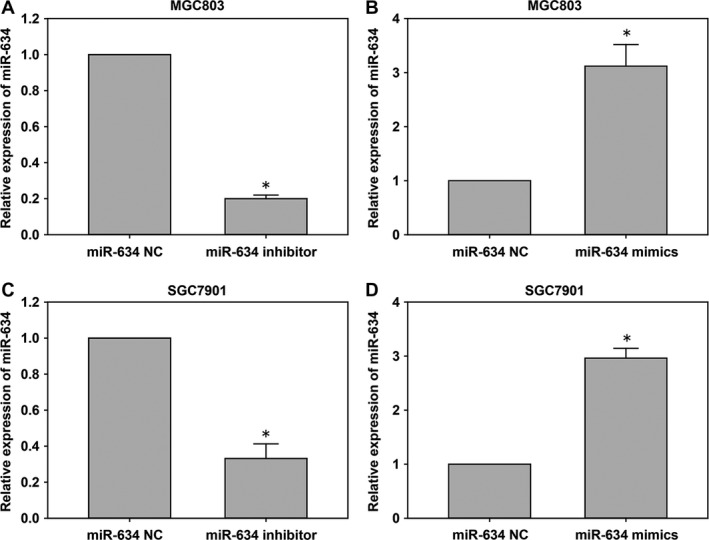
The *miR‐634* expression in cells transfected with *miR‐634* inhibitor and *miR‐634* lentivirus mimics. (A‐D) qRT‐PCR was used to detect the expression of the *miR‐634* in cells after transfected with miR‐634 inhibitor and miR‐634 lentivirus mimics. *, P < 0.05

**Figure 4:**
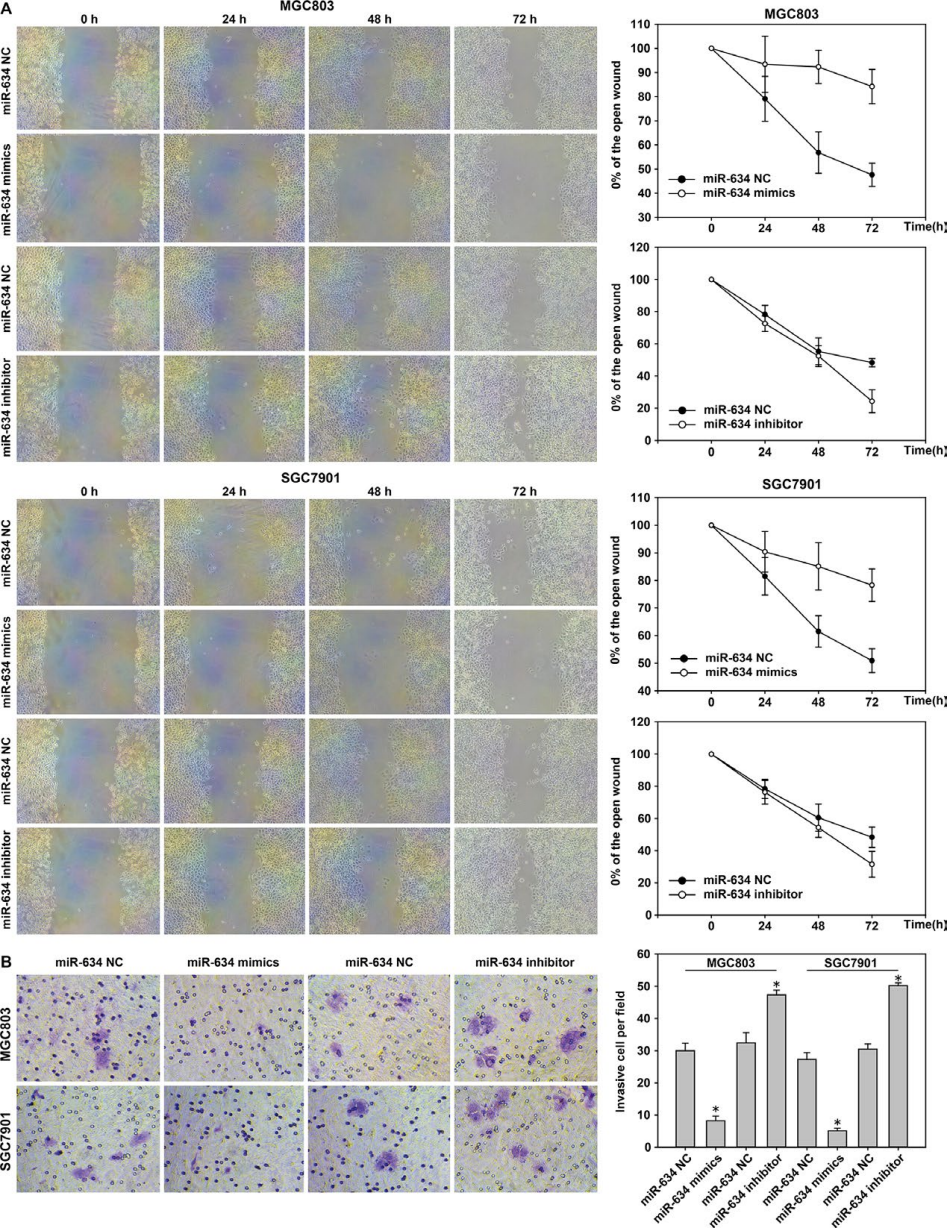
*MiR‐634* suppresses cell migration and invasion in gastric cancer. (A) Wounds were created in MGC‐803 and SGC‐7901 cells after transfection with miR‐634 inhibitor and miR‐634 lentivirus mimics. (B) The Transwell^®^ invasion assay was used in gastric cancer cells transfected with miR‐634 inhibitor and miR‐634 lentivirus mimics. *, P < 0.05

**Figure 5:**
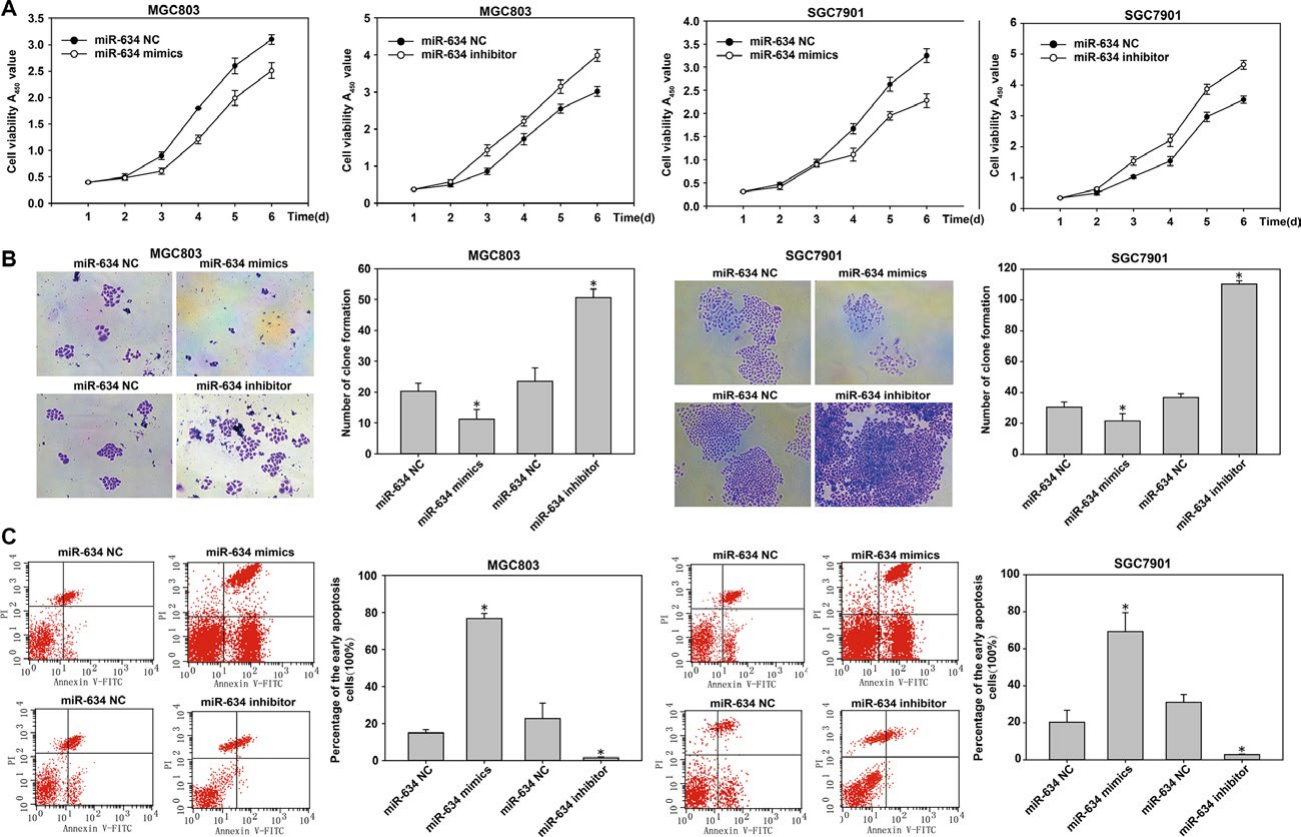
*MiR‐634* suppresses gastric cancer cells proliferation and promotes cell apoptosis. (A) The proliferation of cells transfected with miR‐634 inhibitor and miR‐634 lentivirus mimics was determined using a CCK8 assay. (B) The colony formation results of cells transfected with miR‐634 inhibitor and 634 lentivirus mimics. (C) The cell apoptosis was measured in gastric cancer cells using the Annexin V‐FITC Apoptosis Detection Kit. *, P < 0.05

In order to study the role of miR‐634 in gastric cancer, MGC803 and SGC7901 cells were transfected with miR‐634 inhibitors and mimics based on the results of qRT‐PCR miR‐634 expression in gastric cancer cells. We used qRT‐PCR to verify the effects of the transfections (Fig. 3A–D). The effect of miR‐634 on the migration ability of gastric cancer cells was detected by wound scratch assays. The healing results were observed at 0, 24, 48, and 72 h. The results showed that MGC‐803 and SGC‐7901 cells transfected with miR‐634 mimics inhibited the migration of gastric cancer cells compared with the control group. However, MGC‐803 and SGC‐7901 cells transfected with miR‐634 inhibitor showed the opposite results (Fig. 4A). The effect of miR‐634 on invasion of gastric cancer cells was tested by Transwell^®^ invasion assays. Compared with the control group, MGC‐803 and SGC‐7901 cells transfected with miR‐634 mimics inhibited the invasion of gastric cancer cell lines, whereas MGC‐803 and SGC‐7901 cells transfected with the miR‐634 inhibitor showed the opposite results (Fig. 4B). The effect of miR‐634 on proliferation of gastric cancer cells was measured by the CCK8 assay. Compared with the control group, the growth of MGC‐803 and SGC‐7901 cells transfected with miR‐634 mimics was significantly decreased, while the cells transfected with miR‐634 inhibitor showed the opposite effects (Fig. 5A). Clone formation assays showed that overexpression of miR‐634 inhibited the proliferation of gastric cancer cells, and knockdown of miR‐634 reversed these effects (Fig. 5B).

The annexin V‐FITC apoptosis detection kit was used to analyze the impact of miR‐634 on the function of early gastric cancer cell apoptosis. The results showed that compared with the control group, MGC‐803 and SGC‐7901 cells transfected with miR‐634 mimics showed induced apoptosis in gastric cancer cells. However, MGC‐803 and SGC7901 cells transfected with miR‐634 inhibitors showed the opposite effects (Fig. 5C). In addition, western blot results showed that the expression of phenotype‐associated markers was altered by transfection of miR‐634 mimics or inhibitors. MiR‐634 induction increased the level of the epithelial marker E‐cadherin and suppressed the levels of the proliferation associated markers cyclinD1 and ki‐67. In contrast, miR‐634 inhibition had the complete opposite effect (Fig. 8C and F). Taken together, the results showed that overexpression of miR‐634 inhibited the proliferation of gastric cancer cells in vitro. Overexpression of miR‐634 may therefore play an important role in the migration and invasion of gastric cancer cells.

### High expression of JAG1 in gastric cancer tissues and cells

**Figure 6:**
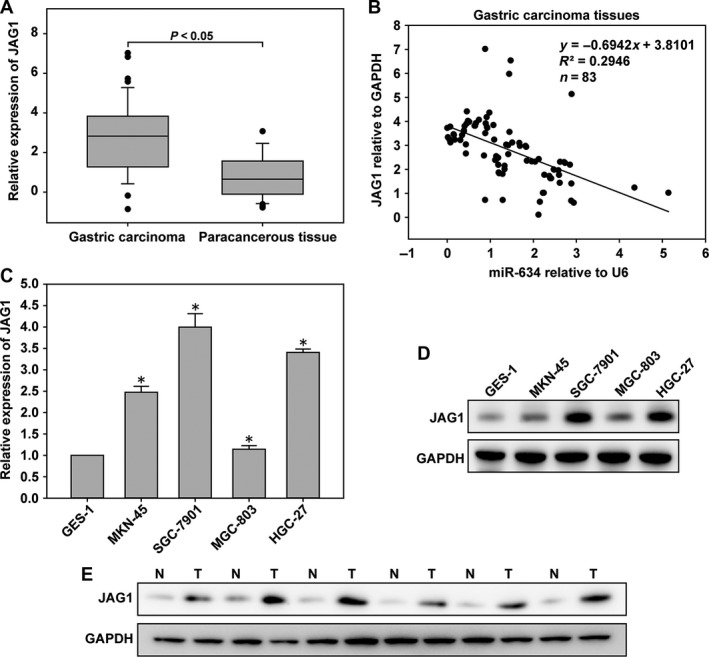
*JAG1* was up‐regulated in GC tissues and cells. (A) The expression level of *JAG1* was determined in 83 pairs of human gastric cancer (GC) tissues and adjacent normal tissues by quantitative real‐time PCR (qRT‐PCR). (B) In human GC tissues, *JAG1* was negatively correlated with miR‐634 at the mRNA level (*n* = 83). (C) The expression level of *JAG1* in GC cells and GES‐1 cells by qRT‐PCR. (D) Western blot analysis of *JAG1* protein expression in GC cells. (E) *JAG1* protein level was examined by western blotting in six paired GC tissues. *, P < 0.05

In order to measure the expression of *JAG1* in gastric cancer, we analyzed the expression of JAG1 in 83 pairs of human gastric cancer and para cancer specimens by qRT‐PCR. The expression level of *JAG1* in gastric carcinoma was upregulated (Fig. 6A), which expression was inversely correlated with miR‐634 in gastric cancer (Fig. 6B). The expression levels of *JAG1* in gastric cancer cell lines and the GES‐1 cell line were measured by qRT‐PCR and western blot methods. The results showed that *JAG1* expression was higher in gastric cancer cell lines compared with the levels in the GES‐1 cell line, as shown in Figure 6C and D. Next, we detected the expression level of *JAG1* protein in six gastric cancer tissues by western blotting. As shown in Figure 6E, the levels of JAG1 protein in gastric cancer tissues were higher than that in adjacent tissues. We also evaluated the clinicopathological features of JAG1. In the gastric cancer tissues, the expression of JAG1 below the median value was selected into low group, while those above the median value was selected into high group. There is no significant association was observed between JAG1 expression and age (*P *=* *0.496), gender (*P *=* *0.794), or stage (*P *=* *1.000). However, its expression was significantly correlated with tumour size (*P *=* *0.026). As shown in Table 1, the expression level of JAG1 was up‐regulated in the tumor size larger than 3 cm group.

### *JAG1* is a direct target of miR‐634

**Figure 7:**
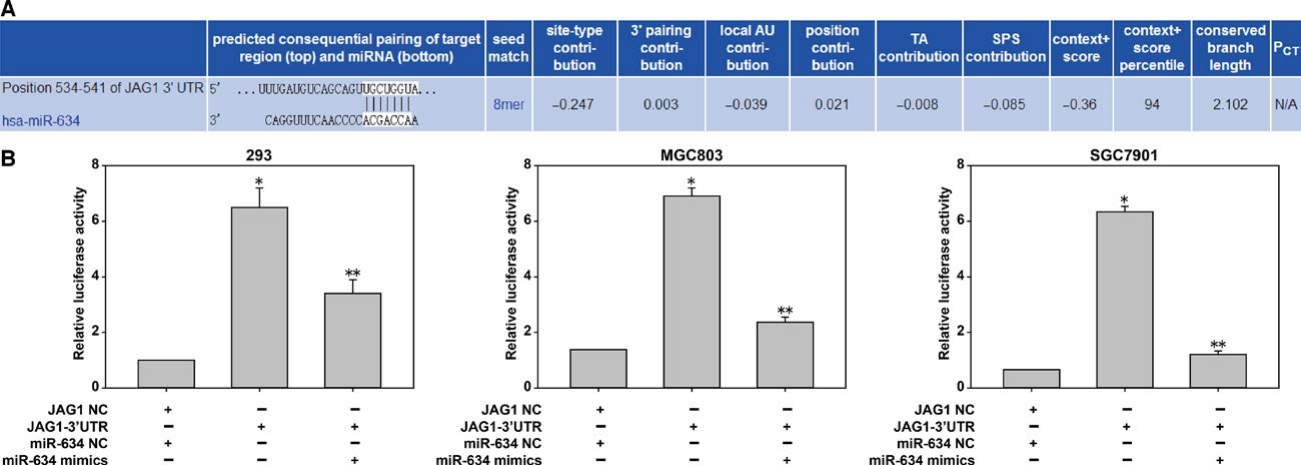
JAG1 was a direct target of miR‐634. The potential miR‐634 binding site at the 3′‐UTR of JAG1 mRNA was computationally predicted by TargetScan. (A) The luciferase activity was analyzed in cells co‐transfected with miR‐634 mimics or negative control cells with pGL3JAG1 or pGL3‐JAG1‐WT. Compared with group JAG1‐NC and miR‐634‐NC and group JAG‐3'UTR, the difference is statistically significant. (*P<0.05) Used group JAG‐3'UTR as control, compared with JAG‐3'UTR and miR‐634‐mimics group, there is statistical significance. (**P<0.05)

**Figure 8:**
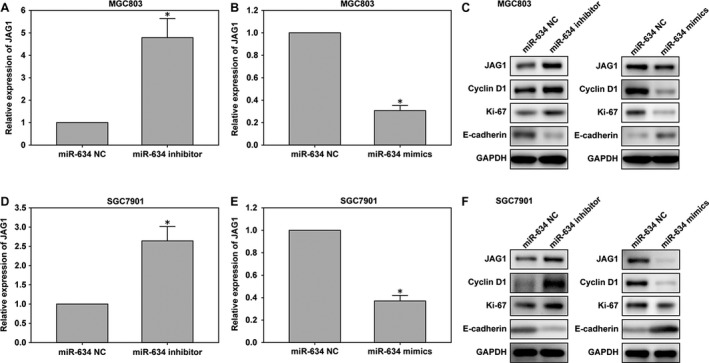
The JAG1 expression in cells transfected with *miR‐634* inhibitor and *miR‐634* mimics lentivirus. (A‐B) The expression of *JAG1* in GC cell lines derived from MGC‐803 cells was determined by quantitative PCR after transfection of *miR‐634* inhibitor or mimics lentivirus. (C) The expression of JAG1, E‐cadherin, and cyclinD1 were detected by western blotting at 48 h after transfection in MGC‐803 cells. (D and E) Quantitative results of *JAG1* after transfection of *miR‐634* inhibitor or mimics lentivirus in SGC‐7901 cells, with *miR‐634* NC as control. (F) The JAG1, E‐cadherin, cyclinD1 and Ki‐67 protein levels were detected in SGC‐7901 cells by western blotting in cells transfected with *miR‐634* inhibitor or mimics lentivirus. *, P < 0.05

MiRNA target prediction sites (miRDB, TargetScan, and miRanda) predicted that *JAG1* may be the target gene of miR‐634 (Fig. 7A). The target gene of miR‐634 was verified by luciferase assays. First, miR‐634 regulation of *JAG1* transcription was demonstrated in 293 cells. Then, in order to further verify the regulatory effect of miR‐634 on *JAG1*, MGC‐803 and SGC‐7901 cells were selected, and the results shown in Fig. 7B suggested that the downstream target of miR‐634 was JAG1. In addition, qRT‐PCR and western blot showed the decreased JAG1 expression after transfection with miR‐634 mimics in MGC‐803 and SGC‐7901 cells (Fig. 8B–F). Moreover, suppression of miR‐634 enhanced the level of JAG1 in the above cells (Fig. 8A, D–F).

### Prognosis of *miR‐634* and *JAG1* in gastric carcinoma

**Figure 9:**
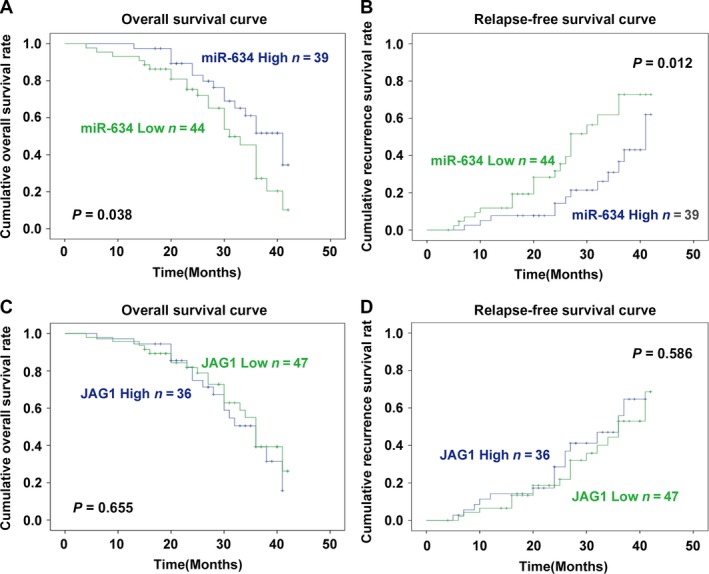
(A) The survival curve for OS of GC patients with different *miR‐634* expression levels (*P* = 0.038). (B) The survival curve for RFS of GC patients with different *miR‐634* expression levels (*P* = 0.012). (C) The survival curve for OS of GC patients with different *JAG1* expression levels (*P* = 0.655). (D) The survival curve for RFS of GC patients with different *JAG1* expression levels (*P* = 0.586).

The recurrence free survival (RFS) was defined as the time from surgery to recurrence or death of any other causes. The overall survival (OS) was defined as the time from operation to death. In the OS analysis, median survival time was 41 months in GC patients with high miR‐634 expression, which was longer than in patients with low miR‐634 expression (31 months, *P *=* *0.038; Fig. 9A). Furthermore, the survival curve showed that the survival time of the elevated JAG1 expression group was lower than that of the JAG1 low expression group, even though there was no statistically difference between them (*P *=* *0.655; Fig. 9C). Recurrence was compared between the elevated miR‐634 and miR‐634 low group in 83 patients. The median follow‐up duration was 28 months (range: 4–42 months). In the RFS analysis, the elevated miR‐634 showed a lower recurrence rate than the miR‐634‐low group (*P *=* *0.012, Fig. 9B). However, there was no difference in RFS between the elevated JAG1 and JAG1‐low groups (*P *=* *0.586, Fig. 9D). These findings suggest that miR‐634 expression might better serve as a prognostic biomarker in GC.

## Discussion

Gastric cancer is one of the most common cancers in the world. Although the treatment of gastric cancer is improving, the treatment of advanced gastric cancer is still not effective. In the past, some reports have suggested that miRNA is a marker of gastric cancer 4, 15. Recent reports have indicated that miRNA can be used as a tumor promoter or as a tumor suppressor, depending on the type of cancer and its target gene 16, 17. For example, in gastric cancer cell lines, miR‐137 played a role in tumor suppression by KLF12 and MYO1C 18. Abnormal expression of miR‐27b inhibited the proliferation of gastric cancer cells 19. MiR‐187 promoted the growth and metastasis of gastric cancer cells by targeting FOXA2 in gastric cancer 16. Further studies have found that a large proportion of the miRNA genes are located close to CpG islands. Therefore, the change of miRNA gene expression may be due to epigenetic regulatory mechanisms, especially the aberrant methylation of CpG islands 20. For example, methylation of the miR‐375 promoter CpG island in esophageal cancer cells led to low expression of miR‐375. However, there was no methylation in normal mucosa 21.

The mechanism of miR‐634 in the carcinogenesis and progression of gastric cancer remains unclear. The purpose of this study was therefore to investigate the expression of miR‐634 in gastric carcinoma and its effects on the function of gastric cancer cells. We found that miR‐634 was downregulated in gastric cancer cell lines and gastric cancer tissues. Clinical data showed that the downregulation of miR‐634 was related to tumor size, and the more miR‐634 expression was downregulated, the larger the tumor volume. Using the online software, Methly Primer, to predict the presence of two CpG islands in the promoter region of miR‐634 gene, it was further speculated that the downregulation of *miR‐634* gene expression may be closely related to the hypermethylation of the promoter region of the gene. The methylation status of the *miR‐634* gene in gastric cancer cell lines was detected by the MSP method for the first time. The expression level of the *miR‐634* gene in gastric cancer cell lines without 5‐aza‐d C treatment was relatively low, and at the same time, the untreated gastric cancer cell lines showed a high methylation status. After 5‐aza‐d C treatment, the expression of the *miR‐634* gene in gastric cancer cell lines increased, and the extent of methylation decreased. The results suggested that DNA methylation may be one of the main mechanisms of downregulation of *miR‐634* gene expression in gastric cancer. We also detected the methylation status of *miR‐634* in 83 gastric cancer tissues and corresponding adjacent tissues. The methylation percentage was significantly higher than that in the corresponding adjacent tissues; also suggesting that *miR‐634* gene expression may be related to the abnormal gene promoter methylation. In addition, studies have shown that miR‐634 expression was downregulated in osteoarthritis chondrocytes 22, hepatocellular carcinoma 23, cervical cancer 24, prostate cancer 25, and ovarian cancer 13, and the expression levels of *miR‐634* were consistent with our findings. We further examined the effects of miR‐634 on gastric cancer cells, and the results showed that overexpression of miR‐634 inhibited gastric cancer cell proliferation, migration, invasion, and apoptosis, and silencing of miR‐634 had the opposite effect. Survival analysis showed that although the survival rate of target genes was no statistical significance, the high expression of miR‐634 could lead to longer survival time and lower recurrence rate. However, the trend of survival in the high expression group was lower than that in the low expression group. Taken together, we reached the conclusion that miR‐634 functioned as anti‐oncogene and suppressed the progression of gastric cancer.

The binding site of *JAG1* indicates that it is a potential target gene of miR‐634. *JAGGED1* (*JAG1*) on chromosome 20, cytogenetic location 20p12.2, chr20:10618331‐10654693, genome mapping (grch37), contains 26 exons of more than 36 kB, and codes for a protein of 1218 amino acids 26. The first discovery of JAG1 associated with cancer was reported in 2005. The study confirmed that the expression of JAG1 is upregulated in human breast cancer and is associated with a poorer overall survival rate in a dose‐dependent manner 27. Our study found an inverse relationship between miR‐634 and JAG1 expression. High expression of miR‐634 inhibited the expression of JAG1, but low expression of miR‐634 promoted the high expression of JAG1. In addition, the high expression of JAG1 was associated with the formation of tumor‐associated angiogenesis in brain and ovarian cancer 28, 29.

There are several limitations in this study. First, the mechanism of JAG1 in gastric cancer is not clear, the role and significance of it in signaling pathway will be further studied. Second, as our collection of samples is limited, it may not adequately reflect overall prognostic characteristics. Third, the follow‐up time was short. Accordingly, we will need a large sample study and a longer follow‐up to verify our results in the future.

In conclusion, this study suggested that aberrant hypermethylation of CpG islands in the promoter region was one of the important mechanisms leading to decreased expression of miR‐634 in gastric cancer. These results provided novel treatment strategies for gastric cancer by modifying the methylation status of the *miR‐634* gene. Through *JAG1*, miR‐634 can downregulate the expression of *JAG1* mRNA. Moreover, this study suggested that miR‐634 is a useful biomarker for early detection of gastric cancer that could improve our understanding of the role of miRNAs in the pathogenesis of gastric cancer and its early diagnosis. In the future, we plan to study the inhibitory mechanism of miR‐634 in gastric cancer to improve early diagnosis and treatment of this disease.

## Conflict of Interest

All authors declare no conflict of interest.
