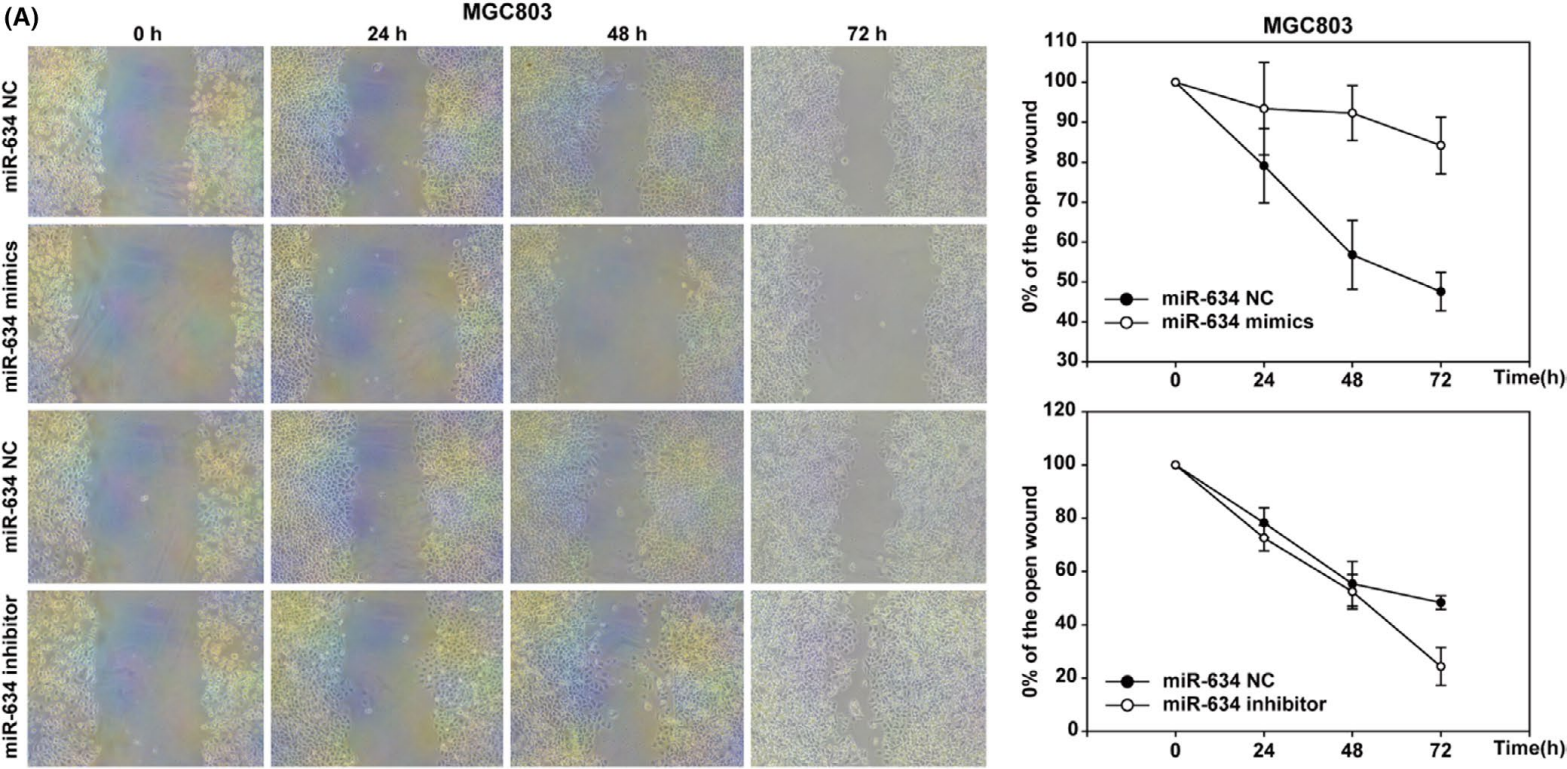# Corrigendum

**DOI:** 10.1002/cam4.4464

**Published:** 2021-11-30

In the article by Jiao et al.,^1^ entitled “Expression of miR‐634 in gastric carcinoma and its effects on proliferation, migration, and invasion of gastric cancer cells,” the authors recently found that the result of the MGC‐803‐miR‐634‐NC in Figure 4A was misapplied during the submission process. Please find the corrected Figure 4A below.

Corrected Figure 4A.